# Practical Notes on Diagnosis and Treatment

**Published:** 1906-06-30

**Authors:** 


### Practical Notes on Diagnosis and Treatment.

### Chrysanthemum in Epilepsy.

A correspondent of the *Lancet* recommends cigarettes made of the dried petals of the chrysanthemum mixed with cascarilla bark in epilepsy. He records some successful results.

### Diabetic Coma.

When the coma is well developed the chance of recovery is very small. But short of this, cases of improvement sometimes recover. Pilocarpin in small and repeated doses and free purgation are indicated, and if the symptoms seem to get worse oxygen should be inhaled.—*Dr. S. West.*

### An Early Symptom of Graves' Disease.

According to Professor Kocher one of the earliest symptoms of Graves' disease, in many cases, is a sudden retraction of the upper lid when the patient is made to look steadily at the physician, or suddenly to look upwards.

### Treatment of Hæmoptysis.

Amyl nitrite, by inhalation in doses of 3 to 9 minims, is advised in hæmoptysis occurring in phthisis pulmonalis. It causes dilatation of the systemic arterioles, and so diminishes the pulmonary blood-pressure.

